# Phosphorylation Codes in IRS-1 and IRS-2 Are Associated with the Activation/Inhibition of Insulin Canonical Signaling Pathways

**DOI:** 10.3390/cimb46010041

**Published:** 2024-01-09

**Authors:** Anabel Martínez Báez, Guadalupe Ayala, Adolfo Pedroza-Saavedra, Hilda M. González-Sánchez, Lilia Chihu Amparan

**Affiliations:** 1 Infection Disease Research Center, National Institute of Public Health, Cuernavaca 62100, Mexico; anymarb@hotmail.com (A.M.B.); gayala@insp.mx (G.A.); apedroza@insp.mx (A.P.-S.); 2 CONAHCYT—Infection Disease Research Center, National Institute of Public Health, Cuernavaca 62100, Mexico; hilda.gonzalez@insp.mx

**Keywords:** IRS-1, IRS-2, insulin, phosphorylation

## Abstract

Insulin receptor substrates 1 and 2 (IRS-1 and IRS-2) are signaling adaptor proteins that participate in canonical pathways, where insulin cascade activation occurs, as well as in non-canonical pathways, in which phosphorylation of substrates is carried out by a diverse array of receptors including integrins, cytokines, steroid hormones, and others. IRS proteins are subject to a spectrum of post-translational modifications essential for their activation, encompassing phosphorylation events in distinct tyrosine, serine, and threonine residues. Tyrosine residue phosphorylation is intricately linked to the activation of the insulin receptor cascade and its interaction with SH2 domains within a spectrum of proteins, including PI3K. Conversely, serine residue phosphorylation assumes a different function, serving to attenuate the effects of insulin. In this review, we have identified over 50 serine residues within IRS-1 that have been reported to undergo phosphorylation orchestrated by a spectrum of kinases, thereby engendering the activation or inhibition of different signaling pathways. Furthermore, we delineate the phosphorylation of over 10 distinct tyrosine residues at IRS-1 or IRS-2 in response to insulin, a process essential for signal transduction and the subsequent activation of PI3K.

## 1. Introduction

Initially, insulin receptor substrates (IRS) were identified as proteins characterized by an approximate molecular mass of 185 kilodaltons (kDa), which exhibited prompt tyrosine phosphorylation upon exposure to insulin within hepatoma cells [1]. IRS-1 was the first member of the family to be identified and cloned [2]. In subsequent investigations, IRS-2 emerged as a phosphoprotein responsive to insulin stimulation, particularly within IRS-1 deficient murine models [3]. It is well known that IRS-1 plays an important role in development, growth, and peripheral insulin responsiveness. Conversely, IRS-2 regulates cerebral growth, body weight maintenance, glucose homeostasis, and reproductive functions [4]. Consequently, IRS proteins have been categorized as active kinase signaling adapter proteins, serving as intermediaries between cell surface receptors and cytoplasmic proteins, including the p85 regulatory subunit of phosphatidylinositol 3-kinase (PI3K), Grb2, and SHP2, among others [5], through the interaction of phosphorylated tyrosines with SH2 domains. The transmembrane receptors endowed with kinase activity responsible for the primary phosphorylation of IRS are the insulin receptor (IR), the insulin-like growth factor-1 receptor (IGF-1R)and the insulin receptor-related receptor (IRR) [6,7,8]. Consequently, this signaling pathway is commonly referred to as the canonical pathway. Moreover, IRS activates non-canonical pathways wherein substrate phosphorylation is orchestrated by cytokine receptors and steroid hormone receptors, including estrogen receptors [9].

**Figure 1:**
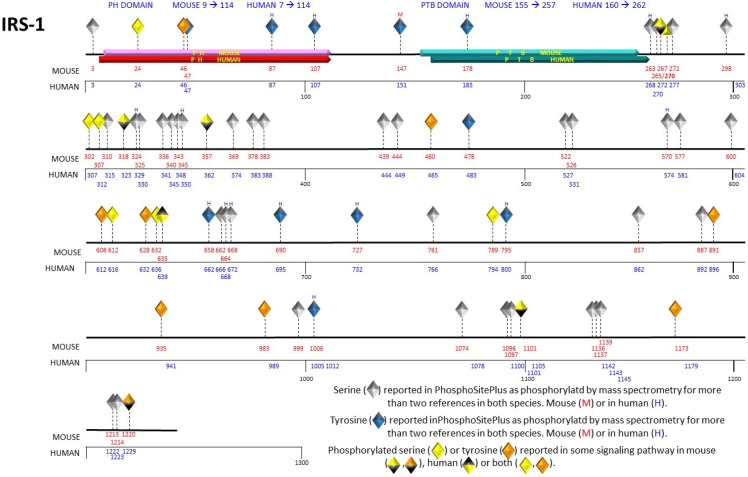
Representation of phosphorylated serine and tyrosine residues in the primary structure of IRS-1. The human and mouse IRS-1 contain the pleckstrin homology (PH) domain, represented in red, and the phosphotyrosine binding (PTB) domain, represented in blue. The serine and tyrosine residues are depicted as diamonds at their respective positions in the primary structure, indicated with red numbers for the mouse and blue numbers for the human. Serine 270 is unique to the mouse sequence. The gray diamonds (serines) and blue diamonds (tyrosines) indicate the phosphorylation-susceptible residues, taking into account those reported in two or more references and identified through mass spectrometry. The orange and yellow diamonds represent the phosphorylated tyrosine and serine residues, respectively.

The IRS protein family consists of six distinct members. IRS-1 and IRS-2 exhibit ubiquitous expression in human tissues, including but not limited to the brain, skeletal muscle, hepatic tissue, cardiac muscle, adipose tissue, renal tissue, ovarian tissue, and mammary glandular tissue [9]. In rodents, the IRS-3 gene is expressed in an active way; however, in the human genome, it is rendered a pseudogene, devoid of functional activity [10]. IRS-4 has been identified in the brain, kidney, thymus, and liver of humans [11]. IRS-5 and IRS-6 are designated as DOK4 and DOK5, respectively. mRNA levels of IRS-5 have been documented in the human renal and hepatic tissues, while mRNA levels of IRS-6 have been found in the muscular tissue [12]. The IRS family proteins exhibit a notable degree of conservation, characterized by the presence of two highly conserved motifs shared among their structural elements. One of these motifs is the pleckstrin homology domain (PH domain), positioned at the amino-terminal terminus, characterized by a composition of positively charged amino acids facilitating protein–cell interactions. The second highly conserved domain is the phosphotyrosine-binding (PTB) domain, responsible for binding to NPXY motifs found on the activated insulin receptor. IRS-2 possesses an additional motif known as the regulatory-loop binding (KRLB) domain, which plays a dual role in facilitating receptor recruitment while concurrently exhibiting reports of inhibitory influence on tyrosine phosphorylation within the IRS-2 molecule [13]. The carboxyl-terminal region, at the distal end, exhibits limited conservation and comprises numerous tyrosine residues susceptible to phosphorylation [14]. The information about the tyrosine and serine residues phosphorylations of was obtained from the PhosphoSitePlus database [15] (Figure 1). Given their extensive presence in human tissues and their prominence as extensively investigated family members, this review will focus its attention on IRS-1 and IRS-2.

IRS-1, a cytoplasmic protein, is encoded by a distinct exon situated on the human chromosome 2q36–37 locus. Comprising a total of 1243 amino acid residues, this protein exhibits an estimated molecular mass of approximately 132 kDa. However, when subjected to polyacrylamide-SDS gel electrophoresis, IRS-1 demonstrates an apparent molecular weight of 185 kDa, which is attributed to its serine phosphorylation status [16]. Conversely, IRS-2 is generated through the transcription of two distinct exons found on the human chromosome 13q34.1 locus. The protein product, comprised of a total of 1322 amino acid residues, possesses a calculated molecular weight of 145 kDa [17].

IRS proteins are susceptible to diverse post-translational modifications, with particular emphasis on phosphorylation events with various tyrosine, serine, and threonine residues. These modifications play a pivotal role in facilitating downstream mediator recruitment and subsequent signal transduction processes. It has been documented that the phosphorylation of specific tyrosine residues, namely, 460, 546, 608, 628, 658, 727, 935, 983, and 1006 within mouse IRS-1, as well as tyrosine residues 536, 594, 649, 734, 814, and 1061 within mouse IRS-2, results in the formation of the YXXM motif. This motif serves as a recognized phosphorylation site by the insulin receptor [18]. Subsequently, the YXXM motif participates in binding interactions with SH2 domains, which are ubiquitously present in several downstream mediators, including the regulatory p85 subunit of PI3K and the Grb2 protein, among others. This interaction cascade culminates in the activation of diverse signaling pathways, notably including the PI3K and mitogen-activated kinase (MAPK) pathways [18].

In this comprehensive review, we meticulously elucidate the roles of phosphorylation events within the intricate signaling pathways governing IRS-1 and IRS-2. We show an unrecognized mechanism of kinase activation and target selection that underlies the generation of disparate cellular responses. Furthermore, we underscore the activation of specific phosphorylation events and their pivotal roles in orchestrating metabolic processes within the cell in response to insulin stimulation. This scrutiny is particularly pertinent in the context of prevalent and adverse metabolic conditions, such as insulin resistance. Moreover, we delineate the intricate pathways leading to non-metabolic cellular processes and expound upon the concurrent activation of diverse intracellular signaling cascades, concomitantly identifying the specific kinases pivotal involved in these multifarious processes. Additionally, we elucidate the range of stimuli to which the IRS-1 and IRS-2 proteins exhibit responsiveness. In addition, our emphasis is directed towards the precise identification of the phosphorylation of tyrosine, serine, and threonine residues in response to insulin or different ligands, subsequently causing the activation or inhibition of cell signaling pathways. Furthermore, we highlight the specific kinases accountable for orchestrating these phosphorylation events. While a substantial portion of the reviewed data is centered on elucidating metabolic responses of the cell to insulin, with a specific focus on prevalent adverse metabolic states like insulin resistance, our examination extends beyond the metabolic realm to encompass phosphorylation events taking place in non-metabolic cellular processes. This broader perspective illuminates the complex interplay of signaling cascades, resulting in the activation of diverse intracellular pathways.

In hepatic cells, FOXO1 and FOXO3a are the main transcriptional factors that regulate the basal expression of IRS-2 at a transcriptional level via an IRE on IRS-2 gene promoter, whereas, in pancreatic β-cells, this regulation is associated with FOXO3a. The stimulation with insulin leads to the inactivation of FOXO1 or FOXO3a, which causes a decreased expression of IRS-2 [19]. It is known that the insulin and IGF2 signaling pathway activates the PI3K/mTOR pathway through IRS-2, which in turn inhibits the expression of IRS2 through feedback [20].

## 2. Phosphorylation Events Targeting Serine Residues on IRS-1

### 2.1. Phosphorylation of Serine Residues on IRS-1 Exerts Inhibitory Effects on the Insulin Signaling Pathway

Serine 307 phosphorylation within IRS-1 represents one of the most extensively documented phosphorylation events. This modification has been widely correlated with the attenuation of or reduction in insulin pathway activity, a phenomenon manifesting in pathological states such as insulin resistance across various physiological contexts, including pregnancy. In a murine bone-marrow-derived cell line, 32D, insulin stimulation promoted phosphorylation at Ser307, orchestrated by the action of the JNK1 kinase. This phosphorylation event impeded the interaction between IRS-1 and the insulin receptor, thereby establishing a mechanistic basis that partially elucidated the development of insulin resistance in the context of obesity [21]. Similarly, within the final stage of gestation in rats, particularly within lumbar adipose tissue cells, a notable augmentation in Ser307 phosphorylation was observed concomitant with a reduction in adiponectin levels. This confluence of events gave rise to a regulatory framework associated with insulin resistance within adipose tissue [22]. Notably, one of the pivotal contributing factors to insulin resistance in the context of obesity and diabetes is the presence of inflammation. Kim and colleagues elucidated that IRS-1 serves as a novel physiological substrate for the mouse Pelle-like kinase (mPLK) in the Fao cell line derived from rat liver [23]. Specifically, they identified that tumor necrosis factor α (TNFα) mediated the phosphorylation of Ser24 within the PH domain of IRS-1, mediated by mPLK in concert with IL-1 receptor-associated kinase (IRAK), constitutes an additional regulatory mechanism that interconnects inflammation signaling with insulin signaling. This discovery holds the potential to contribute to our understanding of insulin resistance etiology. 

The previously mentioned phosphorylation events take place at the amino-terminal end of IRS-1, precisely within the regions housing the remarkably conserved domains inherent to this protein family. Nevertheless, multiple studies have documented the phosphorylation of serine residues within the carboxy-terminal region of IRS-1. Ser1101 residue has been identified as a phosphorylation site on IRS-1 in response to insulin stimulation in L6 myocytes and 3T3-L1 adipocytes. Tremblay and coworkers demonstrated that elevated amino acid availability contributes to insulin resistance, partly through the phosphorylation of IRS-1 at Ser1101 mediated by the S6K1 kinase. In their in vitro assays, S6K1 phosphorylated IRS-1 at Ser1101, and the mutation of serine to alanine at this site largely blocked the ability of amino acids to abolish IRS-1 tyrosine and Akt phosphorylation. In CHO cells expressing the HA-IRS-1, Ser1101 incubated with insulin and amino acids showed increased phosphorylation of Ser1101 compared with the mutant HA-IRS-1 Ser1101Ala, while tyrosine phosphorylation was higher in the mutant. This discovery signifies a causal factor implicated in the onset of insulin resistance during the satiety processes, relevant to both animal and human physiology [24]. On the other hand, DYRK1A is a kinase that phosphorylates serine and tyrosine residues and also reduces insulin resistance. It has been shown that this kinase could phosphorylate IRS-1 and induce a notable increase in Ser312 and Ser616. The Ser312/S616 mutants continued to respond to DYRK1A, indicating that there were other unidentified sites, so more research is needed on the role of IRS-1 phosphorylation and kinase [25].

Numerous studies have consistently elucidated that phosphorylation events occurring on serine residues exert inhibitory effects on the insulin pathway, culminating in the attenuation of insulin signaling and the establishment of a negative feedback loop. However, it is worth noting that alternative findings have suggested that serine phosphorylation plays a role in activation and crosstalk with multiple signaling pathways, each converging at distinct loci to trigger diverse transcription factors and cellular processes. One such pathway that IRS-1 can activate is the mammalian target of rapamycin (mTOR) pathway.

### 2.2. Phosphorylation Events Occurring on IRS-1 That Activate the mTOR Signaling Pathway

It is noteworthy that the mTOR signaling pathway exhibits significant dysregulation when serine residues within IRS-1 undergo phosphorylation. Hence, mTOR occupies a pivotal position in terms of orchestrating the synthesis of proteins, lipids, and nucleotides essential for cellular proliferation and growth. Consequently, it controls the equilibrium between anabolic and catabolic processes in response to prevailing environmental circumstances. As a member of the kinase family related to PI3K, mTOR is a serine and threonine kinase, serving as the catalytic subunit within two discrete complexes designated as complex 1 (mTORC1) and complex 2 (mTORC2). Moreover, mTORC1 drives cellular growth by facilitating a metabolic transition from oxidative phosphorylation to glycolysis, likely enhancing the assimilation of nutrients into the emerging biomass [26].

In mice, Ser307 (equivalent to human IRS-1 Ser312) has additionally been identified as a phosphorylation site on IRS-1, and it has been associated with different signaling cascades, including the mTOR pathway [4,27]. One study demonstrated, in both rat and human pancreatic cells, that glucosamine emulates the stimulatory effects of elevated glucose levels on the kinase activity of JNK and ERK1/2. Furthermore, glucosamine triggers the phosphorylation of IRS-1 at Ser307 and Ser612 residues, respectively. Additionally, the inhibition of JNK and MAPK activity using specific inhibitors resulted in the reversal of phosphorylation at Ser307 and Ser612 residues on IRS-1. This observation suggests that JNK and ERK1/2 are the kinases responsible for phosphorylating these specific residues. These changes are associated with a related reduction in Tyr608 and Tyr628 phosphorylation in two YXXM motifs essential for recruiting the p85 regulatory subunit of PI3K, making the binding of IRS-1 to the p85 subunit, the activation of IRS1-associated PI3K, and the further activation of the Akt/mTOR/PHAS-1/p70S6 kinase protein translation initiation pathway impossible [28]. However, in another article, the serine phosphorylation was found to be a dispensable event; phosphorylation at Ser302 of IRS-1 by S6K kinase was not required to regulate the mTORC1-S6K pathway within the insulin signaling cascade in normal liver tissue [29]. In mice, phosphorylation of Ser307 is often used as an indicator of insulin resistance. Insulin can promote the phosphorylation of Ser307 in IRS-1 through a signal pathway involving PI3K/AKT/mTORC1/S6K1 [30].

Moreover, specific serine residues on IRS-1 can undergo phosphorylation in response to insulin, insulin-like growth factor (IGF), and TNFα, thereby exerting an influence on the mTOR/S6K1 signaling pathway. It has been observed that the phosphorylation of Ser307 in response to TNFα exhibits a correlation with the phosphorylation of JNK, c-Jun, and I-kappa B alpha (IKBα), implying that the phosphorylation of IRS-1 is orchestrated by JNK and IKBα in some cellular contexts. Intriguingly, treatment with aspirin attenuated the phosphorylation of Ser307, along with the phosphorylation of JNK and c-Jun, as well as the degradation of IKBα. Conversely, the phosphorylation of Ser267 and Ser612, induced by TNFα, was associated with the activation of Akt, ERK, PKCξ, and mTOR, and these phosphorylation events were likewise mitigated by aspirin treatment. Hence, it is postulated that aspirin modulates the phosphorylation of serine residues on IRS-1 mediated by multiple kinases [31].

Serine residues 636/639 can undergo phosphorylation by the mTOR kinase in response to diverse stimuli, including insulin and phosphatidic acid, the latter being a product of phospholipase C activity. These phosphorylation events transpire in distinct cellular contexts, such as in mouse adipose tissue cells, where they contribute to adipocyte differentiation [32], as well as in human cell lines, namely, HepG2 (derived from liver cancer) and HEK293 (embryonic kidney). Tzatsos demonstrated that knockdown of mTOR, Raptor, and mLST8, abolished the insulin-stimulated phosphorylation of IRS-1 at Ser636/639 and stabilized IRS-1 after a prolonged period of insulin stimulation, but not Rictor and mSin1 [33]. These results highlight their occurrence in diverse cellular environments.

Additional phosphorylation events documented on serine residues of IRS-1 that could play a role in the activation of the mTOR pathway have been observed at Ser302, Ser422, Ser1101, and Ser632. A comprehensive summary of these phosphorylation sites is presented in Table 1 for reference. Most phosphorylation events documented in databases predominantly occur within the amino-terminal domain of IRS proteins. This specific domain contains the PH and PTB domains, which serve the crucial function of facilitating interactions between IRS proteins and insulin receptors, thereby facilitating the phosphorylation process (Figure 1). The PTB domain exhibits an affinity for binding to phosphorylated Tyr972 and neighboring residues within the juxtamembrane region of the insulin receptor. Simultaneously, the PH domain plays a significant role in both receptor binding and its subsequent stabilization. Moreover, IRS-2 features a domain known as the kinase regulatory-loop binding domain (KRLB). Mutagenesis investigations have underscored the significance of this domain, as it harbors two important tyrosine residues essential for the interaction with the kinase domain of the insulin receptor [34].

### 2.3. Phosphorylation Events Occurring within IRS-1 That Trigger the Activation of the PI3K and MAPK Pathways

The initiation of the MAPK and PI3K signaling cascades represents pivotal pro-cesses in cellular activation and proliferation. It has been established that genes impli-cated in these intricate pathways undergo genetic modifications, rendering them on-cogenic in nature. Consequently, considerable attention has been directed towards the comprehensive investigation of these intracellular signaling cascades within the con-text of neoplastic pathologies, particularly in the realm of cancer research [62].

As previously elucidated, phosphorylation events targeting serine residues within IRS-1 primarily engender an attenuation of the insulin signaling pathway, thereby in-stigating a negative feedback mechanism. Notably, when serine residues are subjected to phosphorylation, they tend to diminish or extinguish the signaling cascade. Howev-er, emerging reports have begun to illuminate an alternative perspective, suggesting that phosphorylation at serine residues may also incite activation of the IRS signaling cascade. This introduces the intriguing notion that serine residue phosphorylation may possess a dual functionality, encompassing signal activation [39] or attenuation [21,37] depending on the phosphorylated serine. It is important to underscore that the manifestation of this duality in serine phosphorylation is contingent upon the intrica-cies of the cellular milieu, warranting further delineation to ascertain the specific cir-cumstances in which activation ensues. It remains to be defined in which cases activa-tion occurs, what can be observed is that the duality of phosphorylations in serine res-idues depends on the cellular context. 

Within hepatocytes, empirical observations have unveiled a noteworthy phe-nomenon, wherein the phosphorylation of IRS-1 at Ser789 by AMP-activated protein kinase (AMPK) promotes insulin-stimulated tyrosine phosphorylation. This mecha-nism stands poised to enable insulin-sensitive cells to dynamically respond to fluctua-tions in the cellular energy milieu, thereby underlining its significance in orchestrating cellular responses to energy status variations. This phenomenon may manifest through the channeling of signaling via IRS-1, potentially culminating in the activation of pathways involved in energy restitution, or by imparting a broader augmentative in-fluence on insulin signaling, facilitated by the upregulation of IRS-1-associated PI3K activity. Exercise is postulated as the instigating factor that sets in motion these un-derlying mechanisms within the musculature. Notably, this study is perceived as pio-neering in its capacity to pinpoint precise serine phosphorylation events within IRS-1 that hold the potential to exert a favorable impact on insulin signaling, thus repre-senting a groundbreaking contribution to the field. However, it remains possible that IRS-1 phosphorylation and enhanced insulin signaling may be independent effects of AMPK activation, so more research is needed on this matter [53].

Several studies suggest that phosphorylation that occurs at a specific serine resi-due within IRS-1 could play a role in the activation of both the PI3K and MAPK sig-naling pathways. Specifically, IRS-1 Ser616 has been identified as susceptible to phos-phorylation induced by 20-hydroxyeicosatetraenoic acid (20-HETE), a bioactive lipid mediator known to elicit endothelial dysfunction. This phosphorylation event is de-pendent on ERK1/2 and leads to impaired insulin-stimulated vasodilator effects that are mediated by the IRS-1/PI3K/AKT/eNOS pathway [51]. Furthermore, it is notewor-thy that Ser312 and Ser616 of IRS-1 undergo phosphorylation in response to angioten-sin II stimulation, a process mediated through the JNK and ERK1/2 signaling cascades in endothelial cells [50].

### 2.4. Phosphorylation of Serine Residues within IRS-1 has the Potential to Activate Distinct and Diverse Intracellular Signaling Pathways

As elucidated throughout this comprehensive review, phosphorylations occurring at various serine residues of IRS-1 exert significant regulatory influence over several essential cellular pathways. These include the mTOR, PI3K, and MAPK pathways. Nevertheless, it is essential to acknowledge that phosphorylation events also involve additional signaling pathways beyond these canonical ones. Furthermore, owing to the dynamic and adaptive nature of cellular physiology, these diverse signaling cascades invariably intersect with the canonical IRS-mediated pathways in response to evolving extracellular signals. The dynamic nature of cellular systems, coupled with the pivotal role of cellular context in governing transcriptional regulation and protein expression, gives rise to the activation of non-canonical signaling pathways. Notably, these path-ways encompass the TNFα pathways and glycogen synthase kinase 3 (GSK3), further emphasizing the multifaceted regulatory landscape within the cell.

Within the TNFα signaling pathway, the kinase S6K1 becomes activated, subse-quently catalyzing the phosphorylation of IRS-1 at several serine residues. In humans, these serine residues include Ser270, Ser307, Ser636, and Ser1101, whereas in rodents, particularly in adipose tissue and liver cells, the corresponding sites of phosphoryla-tion are Ser265, Ser302, Ser632, and Ser1097. This revelation introduces a mechanistic insight into the role of TNFα in eliciting insulin resistance, whereby the activation of S6K kinase serves as a direct mediator responsible for the phosphorylation of IRS-1 [56].

Conversely, within the cascade featuring GSK3 kinase, Ser332 has emerged as a notable substrate of this kinase within IRS-1 [60]. GSK-3 plays a crucial role in the in-sulin signaling pathway [63]. This observation underscores the physiological signifi-cance of Ser332 phosphorylation, elucidating an inhibitory function within the realm of insulin signaling. Phosphorylation of IRS-1 Ser 322 can be considered as a mecha-nism of insulin resistance, point mutations of IRS-1 Ser322Ala or Ser336A prevented GSK-3 phosphorylation of IRS-1. On the contrary, the double mutant IRS-1 Ser322Ala and Ser336Ala considerably increase tyrosine phosphorylation of IRS-1 under both resting and insulin-stimulated conditions [60]. A graphical representation of serine residue phosphorylations on IRS-1 is presented in Figure 1 for visual clarity.

## 3. Phosphorylations of Serine Residues within IRS-2

In contrast to IRS-1, there is less information regarding phosphorylation events at discrete serine residues within IRS-2. The amino-terminal domain of both IRS-1 and IRS-2 exhibits a high degree of conservation; nevertheless, the carboxyl-terminal do-main shows markedly low conservation, except for residues responsible for binding to proteins featuring SH2 domains. The carboxyl terminus constitutes a region charac-terized by an abundance of serine and threonine residues, encompassing several motifs that serve as phosphorylation targets for kinases within the canonical pathway [64]. Serine residues at positions 303, 675, 907, and 1137/1138 within IRS-2 exhibit suscepti-bility to phosphorylation by a diverse array of kinases, including ERK1/2, mTOR, PKC, and PKA. These phosphorylation events play essential roles in regulating numerous cellular processes [41,61,64].

In rat hepatoma cells, the mTOR pathway has been established as intricately linked to the phosphorylation of serine residues within IRS-2, particularly Ser907 and Ser675. Phosphorylation of these residues suggests that PI3K and Grb2 do not partici-pate in this regulatory process. Instead, ERK1/2 and mTOR are potential contributors, likely instigating a negative feedback mechanism [41]. This phosphorylation event was observed in rat hepatoma cells, where a connection between the mTOR pathway and the phosphorylation of IRS-2 serine residues, specifically Ser907 and Ser675, was es-tablished. The phosphorylation of these residues suggests the non-involvement of PI3K and Grb2 in this regulatory process. Instead, it implicates the potential participation of ERK1/2 and mTOR, which may exert a negative feedback mechanism [41]. Neverthe-less, Ser1137/1138 of IRS-2 within mouse kidney cells has been shown to undergo phosphorylation by PKA upon cAMP stimulation, thereby contributing to the stabili-zation of IRS-2 [61]. Similarly, another kinase responsible for phosphorylating serine residues within IRS-2 is PKC. Phosphorylation events have been identified specifically at Serine 303 and 675 sites, and these are induced by the activation of PKC or in re-sponse to Angiotensin II stimulation. The aforementioned phosphorylation events ex-ert inhibitory effects on the tyrosine phosphorylation sites activated by insulin in IRS-2, subsequently impeding the downstream signaling pathways mediated by IRS-2 in endothelial cells. These findings offer valuable insights into alterations in the phos-phorylation of serine residues within IRS-2, a critical component of the insulin signal-ing cascade, which subsequently leads to specific endothelial dysfunction [45]. The phosphorylation events occurring at serine residues within IRS-2 are comprehensively documented in Table 1. It is important to mention that IRS-1 and IRS-2 share 40% of homology in their amino acid sequence. Searching in the sequences of both substrates (IRS-1 and IRS-2) the homologous serine residues that have been reported to be phos-phorylated are Ser632 in IRS-1 and Ser675 in IRS-2 [41].

## 4. Tyrosine Residue Phosphorylation of IRS-1 and IRS-2 Initiates Activation of the PI3K Signaling Pathway

Conversely, phosphorylation events occurring on tyrosine residues within IRS-1 and IRS-2 are intricately linked to the initiation of the canonical insulin signaling pathway. This pathway is initiated upon the binding of insulin to its receptor, leading to receptor activation. Following this sequence, IRS-1 and/or IRS-2 undergo tyrosine phosphorylation, inducing the activation of the PI3K/Akt pathway and thereby facilitating the initiation of insulin-mediated metabolic processes. As previously elucidated, the PI3K kinase emerges as a prominent and extensively studied effector of IRS signaling. Following insulin stimulation, PI3K establishes associations with the phosphorylated tyrosine residues of the IRS-1 and IRS-2 proteins. This molecular interaction catalyzes the generation of phosphatidylinositol-3,4,5-triphosphate (PIP3), which, in turn, orchestrates the activation of phosphoinositide-dependent kinase activity, thus initiating a cascade of events involving downstream effectors including Akt, mTOR, and S6K1. These concerted actions culminate in the facilitation of glucose transport and the synthesis of proteins, integral components of insulin-mediated cellular processes [65].

Tyrosine-phosphorylated IRS proteins exhibit an affinity for Src homology 2 (SH2) domains, which are pervasive in a diverse array of signaling molecules, encompassing Grb2, SHP2, Crk, Fyn, and PI3K, among others. Notably, the PI3K protein comprises two subunits: a catalytic subunit and a regulatory subunit, with the former possessing kinase activity, thereby governing key signaling events downstream. Kinase activity is elicited upon the interaction of specific adaptor proteins, exemplified by IRS-1 and IRS-2, with the regulatory subunit p85. The pivotal mechanism underpinning PI3K cascade activation in response to insulin depends on the binding of p85 [66].

Phosphorylation of tyrosine residues within IRS-1 and IRS-2 has been documented to facilitate their interactions with proteins containing SH2 domains, notably PI3K. In IRS-1, the tyrosine residues identified as key for this association include Tyr460 [67], Tyr608 [68], Tyr628 [69], Tyr891 [70], and Tyr935 [71]. In IRS-2, the sites that have been reported to activate PI3K encompass Tyr649 [72], Tyr671 [72,73], Tyr7 [46], Tyr814 [72], and Tyr970 [73]. Furthermore, it is noteworthy that, within IRS-2, the tyrosine residues exhibiting direct interaction with the phosphorylated insulin receptor encompass Tyr624 and Tyr628 [34], as delineated in Table 2.

On the other hand, Copps and White reported that there is homology in the phosphorylation of tyrosine residues between substrates like Tyr608, andTyr935 in IRS-1, which correspond to Tyr649 and Tyr970, in IRS-2. Tyr608, Tyr649, Tyr935, and Tyr970 serve the function of binding with the p85α subunit [44].

Finally, because few phospho-specific antibodies have been generated, a limited number of phosphorylated residues of IRS-1 and IRS-2 have been analyzed [74,75]. This limits our knowledge about the role that IRS plays in the different signaling pathways activated by insulin.

**Table 2:** Phosphorylated tyrosine residues observed within IRS-1 and IRS-2 upon exposure to insulin, as documented in the Phosphosite database.

IRS Type	Phosphorylated Tyrosine Residue	Characteristics	Reference
IRS-1	46 (mouse)	Does not exhibit an affinity for proteins harboring SH2 domains	[76]
	46 (human)		
IRS-1	460 (mouse)	Demonstrates a low-affinity interaction with the N-terminal SH2 domain of the p85α subunit of PI3K	[67]
	465 (human)		
IRS-1	608 (mouse)	Exhibits the YXXM motif, which serves the function of binding with the p85α subunit of PI3K	[68]
	612 (human)		
IRS-1	628 (mouse)	Plays a crucial role in the activation of PI3K and facilitates the translocation of glucose transporter 4 (GLUT4)	[69]
	632 (human)		
IRS-1	891 (mouse)	Interaction between IRS-1 and SHC	[70]
	896 (human)		
IRS-1	935 (mouse)	The binding site for p85α	[71]
	941 (human)		
IRS-1	983 (mouse)	Recognized and subjected to phosphorylation by the insulin receptor	[77]
	989 (human)		
IRS-1	1173 (mouse)	Binds to tyrosine phosphatase Syp	[77]
	1179 (human)		
IRS-1	1220 (mouse)	Inhibits the interaction between IRS-1 and the SH2 domains of the PTP2C phosphatase	[78]
IRS-2	624 (mouse)	Interacts with the phosphorylated regulatory loop of the insulin receptor	[34]
	628 (human)		
IRS-2	628 (mouse)	Interacts with the phosphorylated regulatory loop of the insulin receptor	[34]
	632 (human)		
IRS-2	649 (mouse)	Essential for its interaction with PI3K	[72]
	653 (human)		
IRS-2	671 (mouse)	Essential for its interaction with PI3K	[72,73]
	675 (human)		
IRS-2	734 (mouse)	Essential for its interaction with PI3K	[72]
	742 (human)		
IRS-2	814(mouse)	Essential for its interaction with PI3K	[72]
	823 (human)		
IRS-2	970 (mouse)	Binding to PI3K	[73]
	978 (human)		

## 5. Conclusions

Phosphorylation events on serine residues within IRS contribute to the mechanisms responsible for signal attenuation within the insulin signaling pathway. These phosphorylations often lead to the reduction or, in certain instances, inhibition of tyrosine phosphorylation, thereby facilitating a negative feedback mechanism. Nonetheless, phosphorylations occurring on these residue types can also function as positive regulators of insulin signaling. Dysregulations in such phosphorylation events are intricately linked to insulin resistance, consequently contributing to the advancement of metabolic disorders, notably diabetes. Importantly, these perturbations can extend their influence to affect various other cellular processes.

Most reported phosphorylations occur in the amino-terminal domain of IRS-1; however, we observed that several authors have reported serine phosphorylations at the carboxy-terminal region.

Moreover, the extensive group of serine and tyrosine residues within IRS that are susceptible to phosphorylation, coupled with the diverse repertoire of kinases capable of targeting these sites, poses a formidable challenge in comprehending the intricate signaling networks orchestrated to establish cellular homeostasis. What is evident is that the biological responses of the cell are dependent upon the actions of kinases and phosphatases, depending upon the specific cellular context.

Serine residues within IRS-1 and IRS-2 adapter proteins exhibit susceptibility to phosphorylation by an extensive spectrum of kinases, thus activating diverse signaling cascades. In contrast, the tyrosine phosphorylation of IRS-1 and IRS-2 adapter proteins is implicated in their direct interaction with the insulin receptor or their engagement with PI3K and other proteins via SH2 domains.

According to the scientific literature, we found sufficient information concerning the phosphorylation of serine residues within IRS-1. Nonetheless, a lack of information exists regarding the comprehensive elucidation of serine phosphorylation events concerning IRS-2. Addressing this knowledge gap is imperative in order to facilitate an integral understanding of the intricate regulatory landscape encompassing serine phosphorylation events within IRS-2.

Accurate knowledge of how different signaling pathways are activated or inhibited by the phosphorylation of different amino acid residues, such as tyrosines or serines, in the ISR-1 or ISR-2 adapter proteins could contribute to the design or application of different drugs, and allow us to propose treatment strategies that could be applied for the control of conditions such as diabetes.

## Figures and Tables

**Table 1:** Serine phosphorylation sites identified within IRS-1 and IRS-2.

Number	Phosphorylation Site	Kinase	Associated Signaling Pathways	Species	Cells/Animals	Stimulus	Reference
Insulin Resistance							
IRS-1	Ser24	mPLK/IRAK	PI3K	Rat	Adipose cells, hepatoma cells	TNF-α	[23]
IRS-1	Ser24	PKC	Insulin signaling	Rat, human	BOSC23 cells and 3T3-L1 preadipocytes	Phorbol 12-myristate 13-acetate	[35]
IRS-1	Ser307	-	Insulin signaling	Rat	Lumbar adipose tissue	Insulin	[22]
IRS-1	Ser307 in rats Ser312 in humans	JNK1	PI3K and MAPK	Rat, human	32D cells	Insulin	[21,36]
IRS-1	Ser307/ Ser632	JNK/IKKβ	PI3K/Akt	Rat	Fao hepatoma cells	H_2_O_2_/insulin	[37]
IRS-1	Ser1101	S6K1	PI3K/Akt	Rat, mouse	L6 myocytes, 3T3-L1 adipocytes, and Fao hepatoma cells	Insulin	[24]
IRS-2	Ser-573	IR, IGF	PI3K/Akt	Human, rat, mouse	Fao and HEK293 cells, livers of mice	Insulin/IGF-1	[38]
**mTOR activation pathway**							
IRS-1	Ser302	ND (mTOR is suggested)	mTOR	Hamster, rat, mouse	Rat muscle tissue cells	Nutrients (amino acids, glucose), insulin	[39]
IRS-1	Ser302	S6K1	PI3K/Akt	Mouse	Liver and primary hepatocytes from mice	Insulin	[29]
IRS-1	Ser307, Ser267, Ser612	JNK, IKK, AKT, ERK, mTOR, PKC-ζ	PI3K-Akt/ mTOR	Human, mouse	3T3-L1 and Hep G2 cells	TNF-α	[31]
IRS-1	Ser307, Ser612	JNK, ERK1/2	Hexosamine pathway, PI3K/Akt/PHAS-1	Rat, human	Human pancreatic islets, RIN1046–38 rat pancreatic β-cells	Insulin, glucosamine	[28]
IRS-1	Ser422	mTORC1	PI3K/Akt	Rat	L6 myoblast cells, HEK293t cells transfected with Myc-IRS-1	IGF	[27]
IRS-1	Ser636/639	mTOR	PI3K/Akt	Human	HEK293 and HepG2 cells	Insulin	[33]
IRS-1	Ser636/639	DEPTOR from mTORC1	PI3K/Akt	Mouse	3T3-L1 cells	C8-PA	[32]
IRS-1	Ser1101	S6K1 is suggested	PI3K-Akt/mTOR-S6K1	Mouse	mHypoE-46 immortalized, clonal, hypothalamic, neuronal cell	Insulin	[40]
IRS-2	Ser675, Ser907	mTOR (Ser675) ERK1/2 and (Ser 907)	mTOR/ERK	Rat	Fao hepatoma cells, HEK 293	Insulin	[41]
**PKC activation pathway**							
IRS-1	Ser307	Shp2-dependent PKC	PI3K/Akt, mTOR, JNK	Mouse/Human	Shp2 knock-out mouse embryonic fibroblasts transfected with human IRS-1	TPA (phorbol ester) and TNFα	[42]
IRS-1	Ser318	PKC-ζ	PKC	Hamster	BHKIR baby hamster kidney cells	Insulin	[43]
IRS-1	Ser357	PKC-δ	PKC	Mouse/human, hamster	C2C12 mouse myoblast cell line and BHKIR (baby hamster kidney cells stably expressing the human insulin receptor)	Insulin and TPA (Phorbol ester)	[44]
IRS-2	Ser303, Ser675	PKC-β	Akt/eNOS	Rat	Zucker obese rats vs. lean rats	Insulin/AngII	[45]
**PI3K and MAPK activation pathways**							
IRS-1	Ser307	JNK	MAPKs (p38 and ERK1/2)	Rat	Skeletal muscle from albino Sprague–Dawley rats	Rats subjected to hindlimb suspension (HLS) to generate atrophic skeletal muscle	[46]
IRS-1	Ser307	JNK	PI3K/Akt, MAPKs	Mouse	3T3-L1 preadipocytes and adipocytes	Insulin, TNF-α	[47]
IRS-1	Ser307, Ser612, Ser632	MEK1, PI3K-mTOR	MAPKs, PI3K/mTOR	Mouse, rat	3T3-L1 adipocytes, L6 myoblast cells transiently transfected with rat IRS-1, skeletal muscle and adipose tissue from Swiss albino male mice	Insulin	[48,49]
IRS-1	Ser312, Ser616	JNK y ERK1/2	PI3K/Akt/eNOS	Human	Human umbilical vein endothelial cells (HUVECs)	Insulin and angiotensin II (AngII)	[50]
IRS-1	Ser616	ERK1/2	ERK1/2 and PI3K/ Akt/eNOS	Human, mouse	Human umbilical vein endothelial cells (HUVECs) and C57BL/6J mice	Insulin and 20-hydroxyeicosatetraenoic acid (20-HETE)	[51]
IRS-1	Ser629	Akt	PI3K/Akt	Hamster, Rat	CHO cells overexpressing human IR and transduced with adenovirus HA-hIRS-1	Insulin	[52]
IRS-1	Ser789	AMPK	PI3K-Akt	Mouse	C2C12 mouse myoblasts fused into myotubes	5-aminoimidazole-4-carboxamide riboside (AICAR)	[53]
IRS-1	Ser794	AMPK	PI3K/Akt, mTORC1/S6K1	Mouse, human	LKB1^-/-^ 3T3 mouse fibroblasts, rat myoblast L6 cell line, and HEK293 cells transfected with *wt*IRS-1	Glucose deprivation, hypoxia, inhibition of ATP synthesis, oxidative stress, 2-deoxyglucose (2-DG)	[54]
IRS-1	Ser1223	Not determined (PKA is suggested)	PI3K/Akt	Hamster/human, rat, human	CHO cells overexpressing human IRS-1, L6 myoblast cells and LE1 cells, and HEK293	Insulin	[55]
**Activation of other signaling pathways**							
IRS-1	Ser270	S6K1	TNF-α/IKK2	Mouse, human	Adipose tissue of obese vs. lean mice, transfected HEK cells	TNF-α	[56]
IRS-1	Ser302, Ser307	JNK1	JNK	Rat/mouse	Fao hepatoma cells, C57BL/6 mouse in vivo	Insulin	[57]
IRS-1	Ser307	IKK	IKK/Myo 1c-NEMO	Mouse	3T3-L1 fibroblasts differentiated into adipocytes	Insulin/TNF-α	[58]
IRS-1	Ser318	PKC-ζ	PI3K/Akt, IR	Human, mouse, rat	Human primary skeletal muscle cells, C2C12 mouse myoblast cell line, L6 myoblast cells	Insulin	[59]
IRS-1	Ser332	GSK3	IR	Rat/human	HEK293 cells transiently transfected with IRS-1 rat gene	Insulin	[60]
IRS-2	Ser1137/ 1138	PKA	PKA	Mouse	HEK293 cells transfected with mouse IRS2	cAMP	[61]
